# The Effect of Coping on the Relationship between Work-Family Conflict and Stress, Anxiety, and Depression

**DOI:** 10.3390/bs14060478

**Published:** 2024-06-06

**Authors:** Cristina de Sousa, João Viseu, Ana Cristina Pimenta, Helena Vinagre, João Ferreira, Rafaela Matavelli, Helena José, Luís Sousa, Fernando Acabado Romana, Olga Valentim

**Affiliations:** 1 Atlântica School of Health, 2730-036 Barcarena, Portugal; hjose@uatlantica.pt (H.J.); lmsousa@uatlantica.pt (L.S.); 2 Atlantic University Institute, 2730-036 Barcarena, Portugal; fromana@uatlantica.pt; 3 Center for Research in Education and Psychology (CIEP-UE), University of Évora, 7005-345 Évora, Portugal; joao.viseu@uevora.pt; 4 Department of Psychology, School of Social Sciences, University of Évora, 7005-345 Évora, Portugal; 5 ISEIT Almada, 2805-059 Almada, Portugal; acrp.pimenta@gmail.com (A.C.P.); maria_helena_santos@iscte-iul.pt (H.V.); 6 ISCTE, Lisbon University Institute, 1649-026 Lisbon, Portugal; 7 Faculty of Medicine, University of Coimbra, 3000-075 Coimbra, Portugal; naquelepordosol@gmail.com; 8 Esucri Faculties, Criciuma 88802-120, Santa Catarina, Brazil; rdmpsiche@hotmail.com; 9 Health Sciences Research Unit: Nursing (UICISA: E), 3004-011 Coimbra, Portugal; 10 Comprehensive Health Research Centre, University of Évora, 7000-801 Évora, Portugal; 11 Nursing School of Lisbon (ESEL), 1600-096 Lisboa, Portugal; 12 Nursing Research, Innovation and Development Centre of Lisbon (CIDNUR), 1600-190 Lisbon, Portugal; 13 CINTESIS&RISE, 4200-319 Porto, Portugal

**Keywords:** coping, work-family conflict, stress, anxiety, depression

## Abstract

The challenges experienced in the context of the pandemic have required a significant reconciliation between work and family domains due to confinement and the need to spend more time at home, which may have increased the levels of stress, anxiety, and depression, making it necessary to use resilient coping strategies to overcome the difficulties felt. This study examined the effect of resilient coping on the relationship between work-family conflict and stress, anxiety, and depression in this context. Data were collected using a self-report protocol from a sample of Portuguese workers (*N* = 476). The results indicated that work-family conflict was positively associated with stress, anxiety, and depression. Resilient coping established a negative relationship with stress, anxiety, and depression. The moderation effect was not corroborated; it was found that in the presence of the moderating variable (resilient coping), the relationship between the variables of work-family conflict and stress, anxiety, and depression was strengthened. This study reinforces the importance of appropriate interventions in resilient coping in the work-family context, which helps control stress, anxiety, and resilience levels.

## 1. Introduction

The safety measures governments worldwide adopted to reduce the impact of COVID-19 included confinement [1]. This measure significantly impacted the labor market, as in the best-case scenario, it had to put its employees to work remotely under unknown (or inadequate) conditions [2]. Thus, workers had to deal with work interference in their family domain. On the other hand, families also had to adapt their routines and needs to this daily functioning. This new situation raised questions regarding the work-family interface. The confinement and the need to spend more time at home working may have contributed to increased work-family conflict (WFC). Some authors [3] found that COVID-19 cognitive job demands were a predictor of WFC due to difficulties separating different domains, i.e., work and family. In situations of high job demands, there is an increase in undesirable work-related outcomes (e.g., stress, anxiety, and depression). According to the JD-R theory [4], personal resources, such as resilient coping, are necessary to decrease the detrimental impact of these demands.

### 1.1. JD-R Theory

This theory has a flexible nature, as it divides work-related characteristics into two broad categories: job demands and job resources [5]. It also has a dynamic nature. According to the JD-R, job demands and job resources activate two processes: (1) health impairment (related to the frequency and magnitude of job demands); job demands deplete employees’ resources (physical, emotional, and cognitive), leading to burnout and, consequently, negative work-related outcomes (e.g., stress, anxiety, and depression) [6,7,8]; and (2) motivational; job resources (e.g., perceived organizational support and performance feedback) foster work engagement, leading, consequently, to positive work-related outcomes [7].

This theory defines specific propositions emphasizing interactions between job demands and resources, personal resources, job crafting, self-undermining, occupational well-being indicators, and work-related outcomes [9]. According to these propositions and the role of the health impairment process, WFC is considered a job demand; resilient coping is integrated into personal resources; and stress, anxiety, and depression are considered negative work-related outcomes [7]. To understand the interactions between these concepts, we first need to understand and define them better.

### 1.2. Work-Family Conflict

This concept is defined by the opposition between roles resulting from the pressures of work and family, which become incompatible, meaning that participation in the work role is hindered by involvement in the family role. This concept has been studied and significantly developed throughout recent years due to the evolution of society and the labor market [10]. According to Bakker and Demeroutti [11], this conflict arises due to excessive demands and difficulties in managing them, which negatively influence well-being or diminish participation in the family context. WFC is considered a job demand in JD-R [12], leading to ill-being. This concept arises when individuals invest in their work tasks while neglecting their duties and involvement in family life. During COVID-19, individuals faced a new challenge: they had to fulfill their work tasks while being involved in their family tasks [13]. According to some authors [14,15,16,17], WFC can be associated with several outcomes as follows: (1) job-related: job satisfaction, burnout, turnover, absenteeism, and organizational citizenship behaviors; and (2) family-related: marital and family satisfaction. In a broader category, outcomes cover life satisfaction, somatic complaints, substance use, or psychological abuse, which can cause stress, depression, and anxiety [17].

### 1.3. Stress, Anxiety, and Depression

These concepts are considered negative work-related outcomes, as described in the JD-R [5,12,18]. Occupational stress arises from the psychological demands of work and the individual’s degree of control over them. It can be divided into physiological (i.e., metabolism changes), psychological (i.e., job dissatisfaction, anxiety, and depression), and behavioral (i.e., changes related to productivity, substance use, and work accidents) categories [19]. Gleitman et al. [20] mentioned that stress occurs in the individual’s body as a mechanism to achieve balance. Sadir et al. [21] argued that the possibility of a negative influence on productivity occurs when the weight of the demands of the environment exceeds the individual’s ability to work. Servino [22] stated that not all individuals wear out in the same way (i.e., with the same stressors); the combination of individual characteristics and the environment will define the occurrence of stress.

The notion of anxiety is typically used to describe an unpleasant emotional state that is used to react to stressful situations. For Sales et al. [23], the notion of anxiety is used to specify an unpleasant emotional state or condition, as well as individual inequalities with a tendency to react to stressful situations. It is essential to consider that states of anxiety are distinguished from trait anxiety since the former presents a short range that becomes evident at a given moment and indicates nervousness and apprehension. Regarding trait anxiety, it is understood as a personality trait with a tendency towards anxiety, determining individual inequalities regarding the method by which the situation is assessed and how one responds to its intensity. The same authors also confirmed that individuals who manifest high trait anxiety have a predisposition to present increased state anxiety [23].

Silva [24] stated that it is difficult to define the notion of depression, as it presents different realities and interpretations. The term depression refers to a symptom that is related to personality types, a mood state, and can be adopted in clinical psychopathology. The notion of depression presents several diagnostic frameworks, representing homogeneous symptoms or severe affective states, depressive personalities, and/or depressive moods. According to Almeida [25], sadness and psychomotor slowing are two traits that characterize depression. The symptoms of depression fall into five categories: emotional, cognitive, motivational, physical, and vegetative [26]. Elsayed et al. [27] referred to studies in which individuals with a high level of occupational stress, less support, and less control were depressed and had decreased levels of physical and mental health.

Consistent with the JD-R, some studies have indicated a positive correlation between WFC and stress and burnout [28] and a positive association between high levels of WFC and high anxiety levels [29]. When employees experience increased job demands, particularly job strain, they can start to present symptoms such as stress, anxiety, and depression [12,30]. Confinement and remote work during the pandemic may have increased job strain, which signals the need to adopt an adaptive coping strategy.

### 1.4. Coping

According to Lazarus and Folkman [31], coping refers to individuals’ cognitive and behavioral efforts to deal with demands when they exceed their resources. The concepts of resilience and coping have been studied as personal resources in the JD-R [12,30,32]. Schaufeli [12] clarified the role of personal resources in this theory. The author argued that it depends on the personal resource studied and suggested that stable personality traits (e.g., optimism) are more likely to act as antecedents of job demands and resources. However, more malleable characteristics (e.g., self-efficacy) could mediate between job demands and well-being [12]. Overall, personal resources are generally linked to resiliency and capture employees’ ability to control and impact their environment in a successful way [33]. According to Sinclair and Wallston [34], resilient coping is how individuals find and overcome the difficulties or limits of a situation presented to them, representing an individual resource. Gadanho [35] defined coping as a behavioral and cognitive effort to endure stressful situations. The coping process can be considered a transfer method between the environment and the individual. This description requires that coping strategies can be learned, applied, and valued, representing a more malleable personal resource moderating between a job demand (WFC) and well-being (low stress, anxiety, and depression). Also, Gadanho [35] stated that coping contains four principles: (1) the relationship between the individual and the environment; (2) control of the stressful situation; (3) how the situation is perceived and cognitively portrayed; and (4) the use of behavioral and cognitive resources to reduce or tolerate their interaction with the environment.

Following these principles, specifically in the work context, employees do not simply react to the environment but actively influence their job demands through adaptive or maladaptive self-regulation strategies [30]. For example, employees who experience increased job strain will follow a self-undermining path, i.e., they will adopt a non-intentional behavioral pattern, creating obstacles because of the strain experienced [36]. This can be interpreted as a maladaptive coping strategy, i.e., inflexible coping. On the contrary, individuals with the ability to use adaptive coping strategies (e.g., flexible coping) will achieve a better adjustment to situational demands [30].

### 1.5. Objectives and Hypotheses

According to previous research, resilience moderates the association between WFC and mental health [37]; individuals with higher resilience deal more adequately with stressors (e.g., WFC) [38]. During COVID-19, some authors emphasized that personal resources (e.g., resilient coping) were favorable, whereas personal demands (e.g., health threats to oneself and others) were unfavorable individual characteristics [39].

In line with this framework, our general objective was to understand the mechanisms that intervene in the relationship between work-family conflict and stress, anxiety, and depression in a pandemic context using a sample of Portuguese workers.

Regarding the specific objectives, they were as follows:

- Analyze how work-family conflict relates to (a) stress, (b) anxiety, and (c) depression.
- Analyze how resilient coping relates to (a) stress, (b) anxiety, and (c) depression.
- Verify whether coping moderates the relationship between work-family conflict and stress, anxiety, and depression.

These objectives were operationalized through the following research hypotheses:

**H1.**  *Work-family conflict is positively related to stress, anxiety, and depression.*

**H2.**  *Coping is negatively related to stress, anxiety, and depression.*

**H3.**  *Coping moderates the relationship between work-family conflict and stress, anxiety, and depression.*

## 2. Materials and Methods

### 2.1. Participants

A sample of 476 Portuguese workers (46.8% females and 53.2% males), with an average age of approximately 43 years old (*M* = 42.66; *SD* = 9.01), was collected. Most participants were married or living in common law (70.8%), had dependent individuals under their responsibility (68.5%), their household was comprised three individuals (*M* = 3.08; *SD* = 1.19; Min. = 1; Max. = 8), and possessed high school education (42.4%).

Regarding employment status, the majority (61.1%) had dependent employment with an open-ended contract and performed their tasks during the COVID-19 pandemic with schedule changes (39.5%).

### 2.2. Measures

Stress, anxiety, and depression were assessed using the Depression Anxiety Stress Scale 21-item version (DASS-21) [26,40]. This measure is composed of 21 items (e.g., I couldn’t seem to experience any positive feeling at all) with a four-point answer scale ranging from 0-Did not apply to me at all to 3-Applied to me very much, or most of the time-Almost always.

The dimension of work-family conflict from the Copenhagen Psychosocial Questionnaire-II (COPSOQ-II) was chosen to assess work-family conflict [41,42]. This dimension is comprised of three items (e.g., Do you feel that your work drains so much of your energy that it has a negative effect on your private life?) with a five-point Likert scale ranging from 1-Never to 5-Always.

The Brief Resilient Coping Scale (BRCS) [34,43] was defined to evaluate resilient coping. This instrument is composed of four items (e.g., I look for creative ways to change difficult situations) with a five-point Likert scale ranging from 1-Does not describe you at all to 5-Describes you well.

Finally, a sociodemographic questionnaire was developed to characterize the sample collected for this study. This questionnaire had questions about gender, age, marital status, household composition, educational background, type of employment contract, and how work tasks were performed during the COVID-19 pandemic.

### 2.3. Data Collection Procedures

The research protocol was developed and hosted on the Google Forms platform, explaining the context and objectives of the study, as well as presenting information on the ethical procedures adopted. The protocol was comprised of self-report measures and a sociodemographic questionnaire. Respondents were informed that the collected data would only serve the research purposes and that they could withdraw their participation at any time. Two inclusion criteria were defined: respondents had to be over 18 years old and be in an active professional situation. The dissemination of the protocol followed a convenience and snowball non-probabilistic sampling technique. Simultaneously, the protocol was disseminated online through social media. Data were collected in the first half of 2023 and referred to the pandemic context.

### 2.4. Data Analysis Procedures

Descriptive (mean and standard deviation values), correlational (Pearson’s correlation coefficient), and reliability (Cronbach’s alpha; α) analyses were performed. These procedures were conducted with the Statistical Package for the Social Sciences (IBM^®^ SPSS^®^) software version 28.

Subsequently, data were assessed through structural equation modeling (SEM) with the Analysis of Moment Structures (AMOS) software version 20. The first step in the analysis was evaluating the multivariate normal distribution. For the maximum likelihood estimation method, skewness and kurtosis values below two (|sk| ≤ 2) and seven (|ku| ≤ 7), respectively, suggest no significant departures from a multivariate normal distribution [44].

Absolute, incremental, and parsimonious fit indices were used to evaluate the overall model fit, following Hair et al. [45] suggestions. The value of the goodness-of-fit Chi-squared test was calculated, which must present *p*-values higher than 0.05. Nevertheless, this index is influenced by the sample size. In samples with several participants, statistically significant values may emerge (*p* < 0.05) [46]. To overcome this gap, other fit indices were adopted: (a) Goodness of fit index (GFI): ≥0.90 indicates a good fit; (b) Root Mean Square Error of Approximation (RMSEA): ≤0.10 indicates an acceptable fit; (c) Standardized Root Mean Square Residual (SRMR): ≤0.08 indicates an acceptable fit; (d) Comparative Fit Index (CFI), Normed Fit Index (NFI), and Tucker–Lewis Index (TLI): ≥0.90 indicates a good fit; and (e) Parsimony Comparative Fit Index (PCFI), Parsimony Normed Fit Index (PNFI), and χ2/df: PCFI and PNFI ≥ 0.60 indicates an acceptable fit and χ2/df ≤ five indicates an acceptable fit [42,43,47,48].

The overall model fit was calculated based on validity and reliability indicators [41]. Factor validity concerns the standardized factor loadings of the items; values equal to or higher than 0.50 must be achieved [43]. Convergent validity was assessed through the Average Variance Extracted (AVE) coefficient; values equal to or higher than 0.50 must be observed [49,50]. Discriminant validity was based on the Fornell and Larcker criterion [51]. Two reliability indicators were selected: Cronbach’s alpha (α) and Composite Reliability (CR); values equal to or higher than 0.70 must be achieved [40].

Data were collected on a single occasion using the same research protocol, increasing the probability of common-method bias. To assess this bias, the unmeasured latent construct method was used [52]. The standardized factor loadings of the two models were compared with or without the common-latent factor. The differences between these models must be lower than 0.200 [53].

Lastly, the structural model was analyzed by observing the sign and statistical significance of the relationships. The moderating effect was based on the matched-pairs method [54]. This method eliminates the gaps of previous methods as it considers the items from the independent and moderating variables to create the interaction effect, and no item can be repeated to form this effect [55]. The items with higher standardized factor loadings must be selected to generate the interaction effect. Moreover, these items must be standardized (Z-values) [54]. The results must report the values of the unstandardized estimates, the results of the *t*-test, and its significance level [54].

## 3. Results

### 3.1. Descriptive Statistics, Correlational Analysis, and Reliability Analysis

Table 1 presents descriptive statistics, correlational analysis, and reliability assessment. Regarding the correlational analysis, statistically significant results were obtained for all associations except for the relationship between resilient coping and work-family conflict. Regarding reliability, the values obtained were all above 0.70.

### 3.2. Overall Model Fit

To respect the assumption of multivariate normal distribution, some indicators had to be removed from the model. After their elimination, it was observed that skewness and kurtosis values respected the threshold values defined by the literature. Thus, it was possible to perform a SEM analysis using the maximum likelihood estimation method. As for the overall model fit, it varied between an acceptable and a very good fit (Table 2).

### 3.3. Measurement Model Fit

Multivariate normal distribution, factor validity, convergent validity, and reliability results are presented in Table 3. It can be argued that there is evidence of factor validity, convergent validity, and reliability.

Table 4 presents the results for discriminant validity. In all situations except one, there was respect for the Fornell and Larcker criterion [51]. The association where this was not registered, between depression and stress, is due to the shared variance that exists between these concepts. Furthermore, the difference between the squared correlation and AVE values can be considered residual.

The evaluation of the common variance, using an unmeasured latent construct, obtained results below 0.200, meaning that the common method variance did not influence the defined model.

### 3.4. Structural Model Fit and Research Hypotheses

Table 5 presents two corroborated research hypotheses (H1 and H2). Work-family conflict established a positive association with stress, anxiety, and depression (H1). In other words, the greater the incompatibility between work and family demands, the greater the incidence of ill-being—in this case, stress, anxiety, and depression. In turn, resilient coping established a negative relationship with stress, anxiety, and depression (H2). In other words, the skills that individuals develop to deal with adverse situations contribute to a reduction in levels of stress, anxiety, and depression. However, the hypotheses related to the moderation effect were not corroborated (H3). It was found that the relationship between the independent and dependent variables was reinforced in the presence of the moderating variable. This aspect is foreseen in the literature [54].

## 4. Discussion

This research analyzed the relationship between work and family domains, two central dimensions in anyone’s life. In 1985, Greenhaus and Beutell defined work-family conflict as a type of conflict that arose when individuals suffered conflicting pressure between several roles, and one of the ways to face this situation was through coping [56]. Thus, the present study sought to add to the existing literature a better understanding of the assessment of the effect of coping in the relationship between work-family conflict and stress, anxiety, and depression in the context of a pandemic. The main objective of this study was to identify the mechanisms that interfere in the relationship between work-family conflict and stress, anxiety, and depression, namely resilient coping, in a pandemic context.

The results obtained through the statistical analysis performed allowed six research hypotheses to be corroborated. Work-family conflict has established a positive association with stress, anxiety, and depression. The greater the incompatibility between work and family domains, the greater the incidence of ill-being. Past studies are in agreement with this result [15]. In turn, resilient coping has established a negative relationship with stress, anxiety, and depression. In other words, the skills that individuals develop to deal with adverse situations contribute to a reduction in levels of stress, anxiety, and depression. Thus, our results are consistent with other studies [35]. Other authors have even demonstrated that different coping strategies correspond to varying levels of stress and depression [27].

However, the hypothesis related to the moderation effect was not corroborated. What was found was that, in the presence of the moderating variable (resilient coping), the relationship between WFC and stress, anxiety, and depression was reinforced. A possible justification for this hypothesis not having been corroborated is that, somehow, the coping mechanisms were ineffective during the pandemic and did not reveal significant associations with the WFC. In exceptional situations of confinement, coping strategies and creative ways of resolving difficult situations do not seem to mitigate the relationship between work-family conflict and psychological effects. On the contrary, they strengthen the relationship between them. What we think may have happened is that the work and family contexts, in a large part of our sample, were interconnected, making it impossible to restrict the extent to which one interfered with the other. This was particularly evident in cases where people had to develop their work and simultaneously meet family demands (e.g., children with online classes). In line with this explanation, several studies have underlined that COVID-19 had a complex effect on both work-family conflict and work-family balance and confirmed that the boundaries between the work and family domains during COVID-19 interfered with each other [57]. This type of situation, along with the experience of confinement, may not have allowed the development of adequate coping strategies to moderate the relationship between WFC and stress, anxiety, and depression. Other studies examined strategies that individuals used to deal with the lack of boundaries between work and nonwork domains in COVID-19 and found difficulties in balancing roles in both domains by constantly thinking about work or by not specifying work and nonwork time [58]. Disregarding occupation, the COVID-19 crisis increased job demands because all employees were required to work differently. It also increased threats to their individual and public health, leading to additional efforts and strain to perform job tasks [39].

Therefore, our results and explanations are consistent with Bakker and de Vries’s [30] ideas that employees are more likely to engage in maladaptive coping strategies, including inflexible coping, when job strain increases. According to these ideas, inflexible coping impairs the ability to adjust to stressors and increases vulnerability to stress, anxiety, and depression [30]. One of the self-regulation strategies advised is recovery, in which individuals try to lower their stress levels during off-job time, engage in other relaxing activities, or distract from work-related issues. Unfortunately, confinement and remote work probably hindered the implementation of these recovery activities. Several studies underlined by Demerrouti and Bakker [39] demonstrated that family adaptation strategies to the COVID-19 crisis differed; some were more collaborative and attentive, while others revealed more sacrifice of their needs or expecting their families to accommodate their job demands. Therefore, the delimitation of boundaries between work and family was an important issue, and coping by restructuring family roles attenuated the work overload of each member for both men and women [39].

## 5. Theoretical and Practical Implications, Limitations, and Suggestions for Future Research

This study has some limitations that need to be acknowledged. Firstly, the research sample is small, which limits the generalization of the results. To overcome this, future research should consider using a larger sample size to enhance the external validity of the findings. Furthermore, using the same research protocol for all participants may have increased the likelihood of common method variance. Moreover, this study followed a cross-sectional design, which prevented causality inference. A limitation may be linked to the application of self-report measures since participants may respond according to normative behavior and social desirability. Another limitation of this study is that the job characteristics of the participants were not assessed. Given the specificities linked to task performance and the work context that can foster or inhibit WFC, this would have been relevant.

Future research should adopt a more robust methodological design, e.g., a longitudinal design, to observe cause-effect relationships and decrease the likelihood of common method variance. Additionally, cross-cultural analysis should be conducted to observe if the results obtained follow the same pattern or if there are differences. Comparative studies between different organizations or countries should also be undertaken to understand the phenomena under analysis. Future research should also expand the range of sociodemographic variables to include variables linked to job characteristics, especially to examine whether distinct job characteristics affect WFC differently.

This study provides valuable insights into the complex interplay between work-family conflict as a job demand and resilient coping as a personal resource in a pandemic context marked by anxiety and uncertainty, revealing that coping strategies should be flexible and allow the mechanism of recovery to deal with the increased strain of job demands. As future directions, the proposition of Demeroutti and Baker [39] makes sense, taking into account our study: regulatory strategies of the family (e.g., boundary management, division of labor) buffer the impact of demands from either domain (i.e., organization, job, home, personal) on health-related outcomes, when they are directed towards providing support or an egalitarian division of labor.

We are currently experiencing social crises characterized by significant uncertainty, as was the case during the pandemic. These adverse contexts increase job demands and the need for family adjustments. Future studies should explore the flexibility or inflexibility of coping strategies to adjust to stressors in these contexts.

## 6. Conclusions

In conclusion, our study sheds light on the intricate interplay between work-family conflict, coping strategies, and mental health outcomes amidst the challenges posed by the pandemic. The findings underscore the significant positive association between work-family conflict and stress, anxiety, and depression, highlighting the impact of increased demands and constrained resources on individual well-being. Practical health interventions directed at individuals are essential for augmenting personal resources to deal with stressful situations (e.g., improving self-monitoring and establishing objectives, reducing ruminations, and improving positive affect). Moreover, our results highlight the crucial role of resilient coping in mitigating the adverse effects of work-family conflict on mental health. The negative relationship observed between resilient coping and stress, anxiety, and depression underscores the importance of fostering adaptive coping mechanisms in navigating through challenging circumstances.

While the hypothesized moderation effect was not supported, it was found that in the presence of the moderating variable (resilient coping), the relationship between work-family conflict and stress, anxiety, and depression was strengthened. This emphasizes the importance of promoting resilience-focused interventions within the work-family context to empower individuals to manage stressors effectively and foster psychological well-being. For instance, coping involves restructuring family roles and the division of labor.

Our findings underscore the importance of implementing targeted interventions to enhance resilience coping strategies among workers, particularly in the face of work-family conflict exacerbated by the pandemic. Therefore, practical interventions derived from organizations are essential and could help workers implement new boundary management strategies to compensate for indistinct physical role boundaries. By equipping individuals, families, and organizations with effective coping mechanisms, policymakers, and public health institutions can contribute to fostering a healthier and more resilient workforce, ultimately promoting individual, family, and organizational well-being in times of adversity. Today’s adverse contexts are different from the pandemic but equally charged with uncertainty and anxiety, and this research may inspire practical interventions in these contexts.

## Figures and Tables

**Table 1:** Descriptive Statistics, Correlational Analysis, and Reliability Assessment (*N* = 476).

Descriptive Statistics			Correlations				
	Mean	SD	Resilient Coping	Work-Family Conflict	Stress	Depression	Anxiety
Resilient Coping	13.28	3.13					
Work-family conflict	2.83	0.917	−0.070				
Stress	4.46	3.98	−0.185 **	0.331 **			
Depression	2.72	3.24	−0.228 **	0.260 **	0.763 **		
Anxiety	1.75	2.80	−0.185 **	0.260 **	0.763 **	0.718 **	

** *p* < 0.001.

**Table 2:** Summary of Global Model Adjustment.

Fit Indices	Results	Comment
χ2	500.173 (*p* = 0.000)	Acceptable
GFI	0.928	Very good
RMSEA	0.054	Good
IC90% RMSEA	[0.049–0.060]	Good
SRMR	0.054	Very good
CFI	0.951	Good
NFI	0.926	Acceptable
TLI	0.943	Acceptable
IFI	0.951	Acceptable
PCFI	0.815	Very good
PNFI	0.794	Good
χ2/df	2.779	Good

**Table 3:** Results of Multivariate Normality, Factor Validity, Convergent Validity, and Factor Validity.

Constructs and Indicators	sk/ku	Standardized Factor Loadings *	Cronbach’s Alpha/CR	AVE
Work-family conflict			0.817/0.891	0.736
1. Do you feel that your work demands a lot of energy that ends up negatively affecting your private life?	−0.404/−0.161	0.898		
2. Do you feel that your work demands a lot of your time, which ends up negatively affecting your private life?	−0.333/−0.227	0.974		
3. Do your family and friends tell you that you work too much?	−0.371/−0.393	0.673		
Resilient Coping			0.798/0.804	0.509
1. I look for creative ways to overcome difficult situations	0.285/−0.491	0.625		
2. Regardless of what might happen to me, I believe I can control my reactions	−0.030/−0.609	0.691		
3. I believe I can grow positively by dealing with difficult situations	−0.060/−0.866	0.796		
4. I actively look for ways to replace the losses I encounter in life	0.142/−0.499	0.730		
Stress			0.911/0.914	0.604
1. I had difficulty calming down	1.206/1.351	0.769		
6. I tended to overreact in certain situations	1.004/0.869	0.667		
8. I felt like I was using a lot of nervous energy	1.085/0.926	0.818		
11. I found myself getting agitated	1.082/1.032	0.851		
12. I found it difficult to relax	0.988/0.577	0.872		
14. I was intolerant of anything that prevented me from finishing what I was doing	1.235/1.315	0.704		
18. I felt that I was sensitive at times	0.719/0.350	0.737		
Anxiety			0.745/0.750	0.600
19. I felt changes in my heart without exercising (e.g., increased heart rate)	1.659/2.263	0.738		
20. I felt scared without having a good reason for it	1.820/3.389	0.810		
Depression			0.741/0.774	0.538
3. I could not feel any positive feelings	1.320/1.422	0.593		
5. I had difficulty taking the initiative to do things	1.267/1.247	0.741		
16. I was not able to be enthusiastic about anything	1.654/2.691	0.845		

* Statistically significant value for *p* < 0.05.

**Table 4:** Discriminant Validity Assessment.

Variables	Resilient Coping	Work-Family Conflict	Stress	Depression	Anxiety
Resilient Coping	**0.509**				
Work-family conflict	0.005	**0.736**			
Stress	0.034	0.110	**0.604**		
Depression	0.052	0.068	0.582	**0.538**	
Anxiety	0.034	0.068	0.582	0.515	**0.600**

Diagonally in bold are the Average Variance Extracted (AVE) values.

**Table 5:** Structural Model Results.

Research Hypotheses	Non-Standard Estimates	*t*-Test Values	Hypothesis
H1:CTF → Stress	0.158	5.871 ***	Confirmed
CTF → Anxiety	0.128	4.650 ***	Confirmed
CTF → Depression	0.051	2.179 *	Confirmed
H2: CR → Stress	−0.148	−4.522 ***	Confirmed
CR → Anxiety	−0.150	−4.393 ***	Confirmed
CR → Depression	−0.175	−5.623 ***	Confirmed
**Moderation analysis**			
H3: CTF*CR → Stress	4.352	2.607 **	Unconfirmed
CTF*CR → Anxiety	3.834	2.609 **	Unconfirmed
CTF*CR → Depression	3.169	2.583 **	Unconfirmed

CTF = work-family conflict; CR = Resilient Coping; * *p* < 0.05; ** *p* < 0.01; *** *p* < 0.001.

## Data Availability

Data are available upon request to the corresponding author.
